# Learning and replaying spatiotemporal sequences: A replication study

**DOI:** 10.3389/fnint.2022.974177

**Published:** 2022-10-14

**Authors:** Jette Oberländer, Younes Bouhadjar, Abigail Morrison

**Affiliations:** ^1^Institute of Neuroscience and Medicine (INM-6), Institute for Advanced Simulation (IAS-6), JARA-Institute Brain Structure-Function Relationship (JBI-1/INM-10), Research Centre Jülich, Jülich, Germany; ^2^Department of Computer Science 3-Software Engineering, RWTH Aachen University, Aachen, Germany; ^3^Jülich Research Centre and JARA, Peter Grünberg Institute (PGI-7, 10), Jülich, Germany; ^4^RWTH Aachen University, Aachen, Germany

**Keywords:** timescales, sequential dynamics, spiking networks, spatiotemporal sequences, synaptic plasticity, recurrent network, replication

## Abstract

Learning and replaying spatiotemporal sequences are fundamental computations performed by the brain and specifically the neocortex. These features are critical for a wide variety of cognitive functions, including sensory perception and the execution of motor and language skills. Although several computational models demonstrate this capability, many are either hard to reconcile with biological findings or have limited functionality. To address this gap, a recent study proposed a biologically plausible model based on a spiking recurrent neural network supplemented with read-out neurons. After learning, the recurrent network develops precise switching dynamics by successively activating and deactivating small groups of neurons. The read-out neurons are trained to respond to particular groups and can thereby reproduce the learned sequence. For the model to serve as the basis for further research, it is important to determine its replicability. In this Brief Report, we give a detailed description of the model and identify missing details, inconsistencies or errors in or between the original paper and its reference implementation. We re-implement the full model in the neural simulator NEST in conjunction with the NESTML modeling language and confirm the main findings of the original work.

## 1. Introduction

The ability to learn sequences is essential for a wide range of tasks such as motor production, language processing, and high-level cognitive processes including planning and reasoning. To acquire such skills, the brain processes spatiotemporal sequences (Dehaene et al., 2015; Henin et al., 2021) involving multiple regions such as the neocortex and the hippocampus (Xu et al., 2012; Gavornik and Bear, 2014). Depending on the nature of stimuli to be processed, learning sequences often entails integration of multiple timescales; neurons typically operate at millisecond timescales, whereas behavioral timescales can range from a few milliseconds to hundreds of milliseconds or longer (Edwards et al., 2002; Mauk and Buonomano, 2004; Schirmer, 2004). Thus, bridging substantially different timescales is a crucial component of sequence learning in the brain.

The underlying neuronal mechanisms for learning spatiotemporal sequences remain largely unknown. Machine learning techniques such as artificial recurrent neural networks (Hochreiter and Schmidhuber, 1997; Schuster and Paliwal, 1997) can successfully handle tasks such as speech recognition, translation, and text generation, but they achieve these with no clear biological interpretation. Networks composed of biologically plausible components rely mostly on prewired connections to establish desired dynamics (Setareh et al., 2018). Others learn the temporal order without learning element specific timing and duration (Bouhadjar et al., 2022).

The model proposed by Maes et al. (2020) represents an interesting hypothesis with regard to neural sequence processing, as it can learn, store, and replay spatiotemporal sequences using biologically inspired models of neurons, synapses, and learning rules. The model uses two components to learn the sequences: a recurrent neural network (RNN) and a read-out layer. The temporal information is captured by the RNN, which consists of clusters of excitatory neurons. After learning, cluster *i* develops strong connections to cluster *i*+1, but weak connections to *i*−1. The learned connectivity and randomly generated external input (i. e. spontaneous input) cause the recurrent network to exhibit sequential behavior, with clusters becoming active one by one. Effectively, a *neural clock* is created. Each cluster is active not only over the intrinsic timescale of neurons and synapses (~1 ms) but at larger timescales relevant to the behavior (~15 ms and up). The clock then drives the read-out neurons activating different spatial information. The model can learn complex, higher-order sequences in parallel and is capable of learning highly variable spatial dimensions: from simple sequences of letters such as *ABCBA* to frequency spectra pertaining to the song of a bird.

In this Brief Report within a special issue on Reproducibility in Neuroscience, we present a replication of the original study by Maes et al. (2020). We hereby restrict our analysis to the replicability of the original study and do not consider robustness to parameter settings or performance aspects. Here, we use the term *replication* in the R^5^ sense described by Benureau and Rougier (2018), i.e., striving to obtain the same results using an independent code base, whereas a *reproduction* (R^3^) of the model entails re-running the original code. These terms are used by some authors the other way around: see Plesser (2018) for an overview and analysis. In general, replicating a study is an excellent method for locating any hidden assumptions or errors in the original implementation, far more so than examining or re-running the original code. However, a replication crisis has become prevalent in most fields of science, with a significant proportion of empirical results being difficult if not impossible to replicate (Baker, 2016). Aided by the description in the original paper and by instrumentation of the original code in *Julia* and *MATLAB*, we re-implement the model using the open source software *NEST* (Hahne et al., 2021) to simulate the network, *NESTML* (Babu et al., 2021) to define the inhibitory neurons and Python for data analysis. Access to the original code, but also to our re-implementation, can be found below in the Data Availability Statement.

Our results confirm the original findings. However, we also identified discrepancies between the paper and the code and repair numerical errors, and thereby provide an accessible and maintainable implementation of the model which is more consistent with its description in text and tables and contains fewer errors. The model is therefore on a substantially better footing to serve as a basis for future extensions to address new problems or account for more diverse experimental data.

## 2. Results

Maes et al. (2020) decompose the learning of spatiotemporal sequences by training the timing component and the target sequence separately. Time is discretized by sequential activation of excitatory clusters that are recurrently connected. Each cluster in the recurrent network represents a time interval and the cyclical activation of the clusters constitutes a neural clock. Once the training of the clock is complete, it drives a read-out layer to enable the learning of the target sequence. Each neuron in the read-out layer represents a distinct element of the sequence. By alternate activation of these neurons in proper order, the sequence can be replayed. In general, the learning is enabled by allowing modifications of connections within the RNN and between the RNN and the read-out layer. The model is described in detail in 4. In addition, a detailed model table and all corresponding parameter values can be found in the Supplementary materials.

### 2.1. Encoding discrete time with a recurrent network

**Figure 1:**
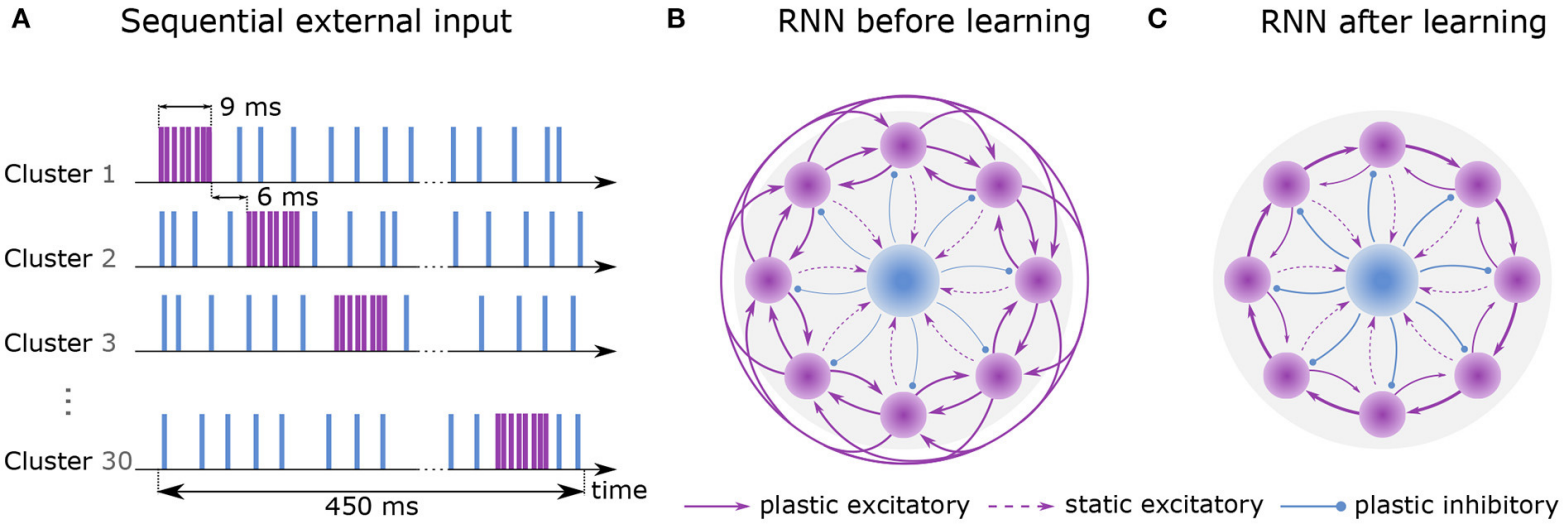
Learning sequential dynamics. **(A)** External spike trains are sent to every excitatory neuron during the first hour of learning (sequential input). Excitatory and inhibitory stimulus are depicted in purple and blue, respectively. Each cluster is excited sequentially for 9ms at a rate of 22.5k spks/sec (purple) and inhibited at a rate of 4.5k spks/sec otherwise (blue). The activation of the next cluster is preceded by 6 ms. Here, one round of sequential input takes 450 ms, which is dependent on the number of clusters (*N*^C^ = 30). **(B)** Recurrent network before learning. Purple circles represent excitatory clusters, each consisting of 80 excitatory neurons. For visual clarity, only 8 clusters are shown rather than 30. Blue circle represents 600 inhibitory neurons. Dashed arrows represent static connections; solid arrows represent plastic connections. Every connection of a particular connection type has the same synaptic weight. **(C)** After 2 hours of training, the recurrent network develops strong connections between cluster *i* and cluster *i*+1 and weaker connections between cluster *i* and cluster *i*−1. Connections to other clusters are vanishingly small.

The recurrent network consists of *N*^E^ excitatory neurons which are divided into *N*^C^ clusters of equal size. Its purpose is to discretize the flow of continuous time by successive activation of the clusters. Before training, all synaptic weights between excitatory neurons have identical strengths (Figure 1B). Due to a voltage-based spike-timing-dependent plasticity (STDP) rule, the strength of connections increases 1) between the excitatory neurons of the same cluster and 2) between the neurons of cluster *i* to cluster *i*+1 forming a feedforward structure (Figure 1C). To avoid runaway dynamics (Chen et al., 2013), a central inhibitory population of *N*^I^ inhibitory neurons is sparsely connected to all excitatory clusters. Their synapses follow a symmetric STDP rule with constant depression.

Training is divided into two phases. First, an external Poisson process stimulates the clusters one by one for 9 ms each. Stimulation of the next cluster is preceded by a 6 ms gap. When a cluster does not receive excitatory input, it receives inhibitory input (see Figure 1A). Once the last cluster has been stimulated, the first cluster is stimulated again, and the procedure is repeated continuously for 1 hour of biological time. Throughout the entire phase, the inhibitory neurons constantly receive external excitatory input. In the second phase, external Poisson processes emit spikes randomly and without any structure to all neurons for an additional hour but with different rates for inhibitory and excitatory neurons. For a full description of the learning protocol, please refer to 4.

**Figure 2:**
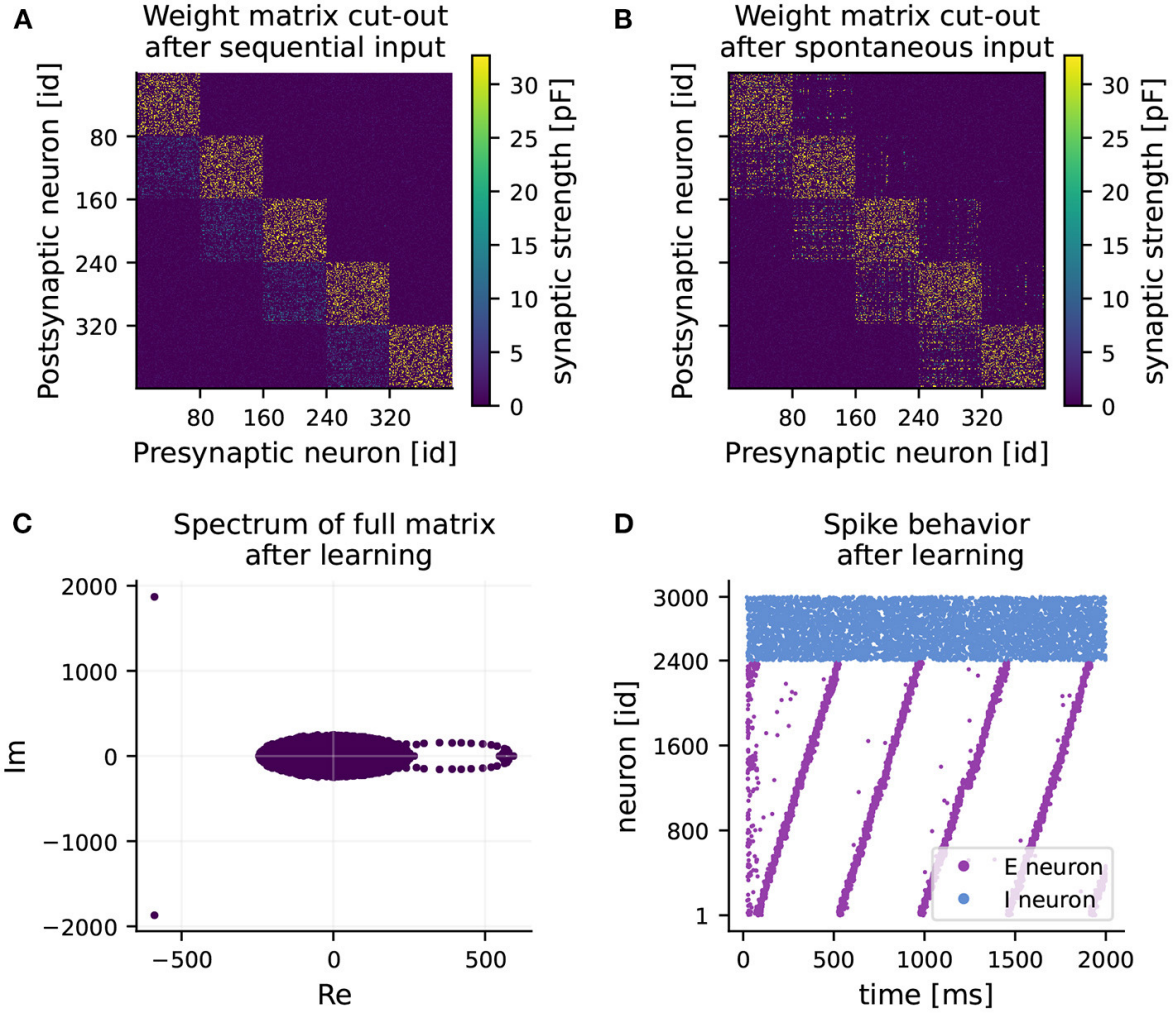
RNN learns feedforward structure and develops sequential dynamics. **(A)** Weight matrix of connections from excitatory to excitatory neurons (EE) after 1 hour of training (sequential input only). Note, only a 400 × 400 sub-matrix including five clusters is shown. **(B)** Final weight matrix of EE connections after 2 hours of training (sequential input followed by spontaneous input). **(C)** Final spectrum of the full weight matrix. The majority of the eigenvalues are distributed in a circle around the origin (appearing as an ellipse due to different scaling of the axes). **(D)** Spiking activity of excitatory (purple) and inhibitory (blue) neurons over time after training under spontaneous external input, while keeping the learned weight matrix frozen.

After 1 hour of learning, strong intra-cluster connections are formed (see Figure 2A). Also, weights from cluster *i* to cluster *i*+1 increase slightly, while all other connections are weakened. In the second hour of learning, mainly connections of one cluster to its adjacent clusters experience potentiation (see Figure 2B). Weights on the main block diagonal, i.e., intra-cluster connections undergo predominant but slight depression. The learned feedforward structure reveals itself as a ring-shaped pattern of eigenvalues, known as leading eigenvalues, in the right half of the spectrum of the full weight matrix (see Figure 2C). Besides the leading eigenvalues, most eigenvalues are distributed inside a circle around the origin in the complex plane. This phenomenon is known in random matrix theory as the circular law (Tao and Vu, 2008), and has also been observed in matrices of synaptic weights (Rajan and Abbott, 2006). The third characteristic of the spectrum is the pair of eigenvalues with large negative real part. They provide information about the balance of the network in terms of excitation and inhibition (see Maes et al., 2020).

At the end of the second phase, the network develops fully coherent sequential dynamics when receiving spontaneous input (see Figure 2D). The clusters become active one after the other with a seamless transition. Eventually, the recurrent network operates as a neuronal clock with a period of 470 ms on average, matching approximately the duration of one round of sequential stimulus (450 ms). A longer activation interval and lower spike density can be observed at the activation of the 15th cluster. This minor quantitative disparity from the original results can be explained by our reduced normalization frequency of every 450 ms, chosen to reduce simulation times, rather than every 20 ms as in Maes et al. (2020).

The learned weight matrix and the corresponding spectrum exhibit a connection structure consistent with the one of Maes et al. (2020). Hence, we can confirm the main finding that strong intra-cluster connections and feedforward structures are established, eventually resulting in sequential dynamics in the recurrent network with no emergent pathological behavior.

### 2.2. Learning a higher-order sequence

Learning higher-order sequences is of particular relevance, since many real-world spatiotemporal sequences have non-Markovian properties, i.e., recalling the next element of a sequence depends on the current and previous elements. As an example, consider the sequence *ABCBA*, where the transition from *B* to the next element cannot be determined by the current element alone, since there is a transition from *B* to *A* as well as from *B* to *C*. Typically, knowledge of the past is required, which makes it harder to learn.

The model proposed by Maes et al. (2020) circumvents this issue by learning the clock prior to using any spatial information. Each element from the alphabet can then easily be assigned to multiple moments in time and thus positions in the sequence, by learning the appropriate connections between the clusters in the recurrent network and the read-out neurons.

**Figure 3:**
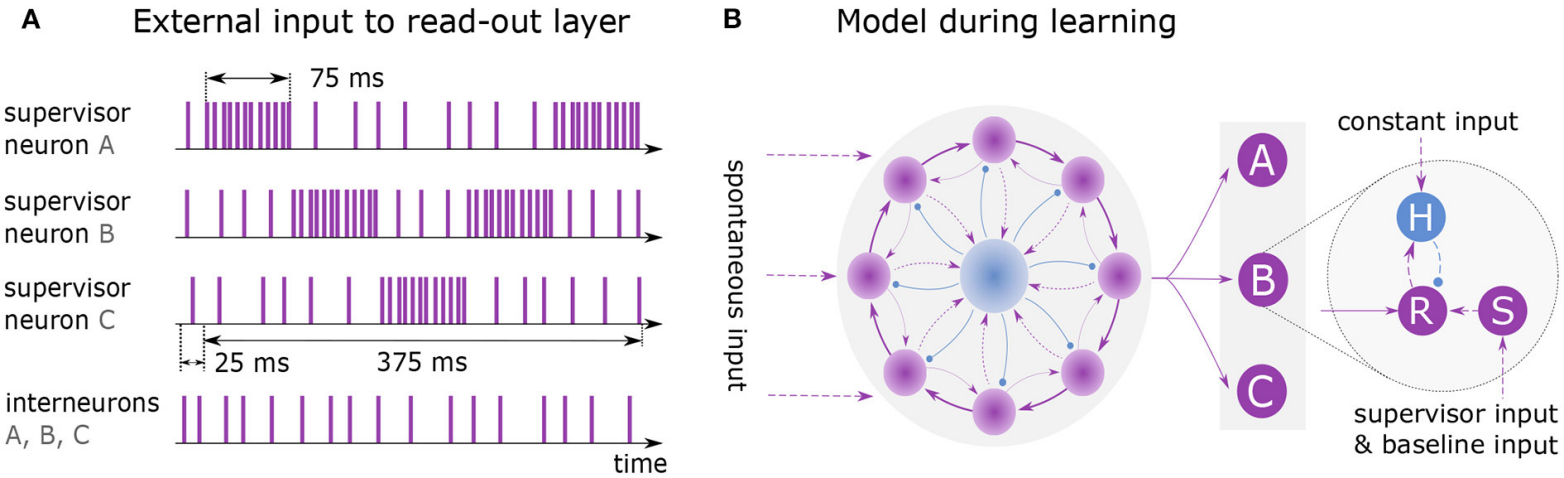
Learning and replaying a sequence. **(A)** External spike trains to supervisor neurons and interneurons during learning of sequence *ABCBA*. A single presentation is shown with total duration of 400 ms including 25 ms lead time. Each element is presented for 75 ms by stimulating the associated supervisor neuron with rate 10k spks/sec. When an element is not presented, its supervisor neuron receives input at baseline rate 1k spks/sec. **(B)** Model architecture while learning the sequence. Clock is all-to-all connected to the read-out layer. Units *A, B* and *C* each consist of three neurons: read-out (R), supervisor (S) and interneuron (H).

Figure 3 illustrates the training and architecture of a network learning the sequence *ABCBA* composed of three elements (*A, B, C*). The excitatory neurons of the clock network are all-to-all connected to a read-out layer, following the voltage-based STDP rule. The layer consists of several independent read-out neurons, where each neuron is associated with a distinct element of the target sequence. During learning, each read-out neuron is connected to one supervisor neuron and one interneuron (see Figure 3B).

Before the sequence is learned, the network must reach a state where it reliably exhibits sequential dynamics. This is achieved by running the simulation for 50 ms, with only the neurons in the recurrent network receiving spontaneous input ($r_{\text{exc}}^{\text{I}}$ and $r_{\text{exc}2}^{\text{E}}$). Afterward, the elements of the sequence are presented to the network by exciting the associated supervisor neurons for 75 ms each, using a Poisson input (Figure 3A). The sequence is repeatedly shown to the network for 12 seconds. In each round the sequence must be presented at the same time given by the clock. Note that the presentation time of a sequence may not exceed the duration of a clock cycle.

**Figure 4:**
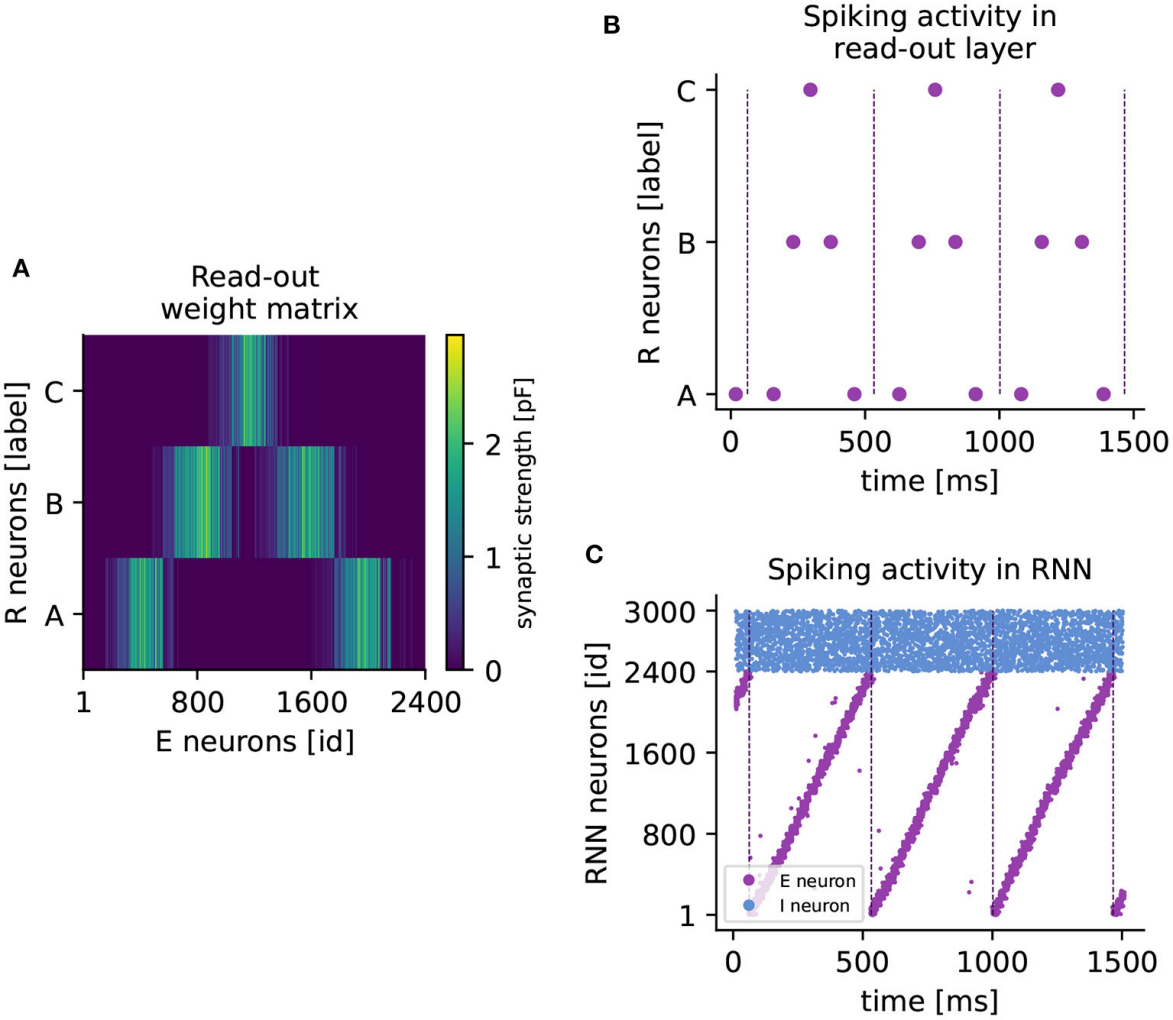
Learning a higher-order sequence. **(A)** Weight matrix of connections from excitatory to read-out neurons (RE) after sequence *ABCBA* has been learned. Each read-out neuron is represented by its associated element (*A,B,C*). The colorbar indicates the synaptic weight. **(B)** Spiking activity of read-out neurons after learning the sequence *ABCBA*. Read-out neurons receive only input from the clock network. Dashed vertical lines mark the end (or the beginning) of a period of the clock. **(C)** Spiking activity of neurons in the RNN under spontaneous external input after training. Purple (blue) dots represent spikes of excitatory (inhibitory) neurons. Dashed vertical lines mark the end of each clock period.

When a cluster is active at the same time as a read-out neuron, the voltage-based STDP rule potentiates the connections between them. The network approximates the element-specific presentation times of a sequence by encoding them in the synaptic strength of read-out connections (see Figure 4A).

After learning, the sequence can be replayed by spontaneous input to the RNN only. The replay is shown in Figure 4B, where the read-out neurons spike in the correct temporal order, synchronized with the clock dynamics (Figure 4C). The replay never stops, as the clock is active in a continuous loop. Maes et al. (2020) report that for each element, the read-out neuron spikes twice on average. In our hands, each read-out neuron reliably produces one spike per element. However, the key finding that the network can learn higher-order sequences is confirmed.

## 3. Discussion

### 3.1. Summary

We have successfully replicated the key findings of Maes et al. (2020) with only minor quantitative deviations. The recurrent network discretizes the time by successive activation of the neuronal clusters. The desired sequential dynamics are reliably reproduced under spontaneous input. Using this temporal backbone, a higher-order sequence such as *ABCBA* can be learned and stored in the read-out connections between the RNN and the read-out layer.

In the replication process, we have followed as closely as possible the parameters, learning protocols, and architectures of the original work. However, replicating a model raises many challenges. Parameters may be missing, unspecified, or inconsistent. Equations may be stated incorrectly, or certain assumptions may not have been mentioned. Implementation errors in the original source code may, in the worst case, fundamentally change the dynamics of the network, but even if they do not, they prolong the replication process. We are able to resolve many of these issues, which we summarize below, through examination and instrumentation of the source code provided by Maes et al. (2020). However, even after having fixed the implementation errors, the two codes produce similar results but are not identical. We find it likely that the numerical disparity is largely due to differences in the implementation frameworks. NEST provides an event-based approach for weight updates, which are performed only after the pre-synaptic neuron has spiked. The implementation in Julia and MATLAB is time-based and weight updates are constantly performed at each simulation step. Furthermore, different random number generators and numerical integration methods for solving differential equations prevent a one-to-one comparison of simulation results; exact spike-correspondence is not an achievable aim (but see Pauli et al., 2018). As the key findings of Maes et al. (2020) are formulated in terms of network dynamics, changes in weight between populations, and functionality, rather than specific spike times, we conclude that these disparities do not detract from the result, and our implementation can be considered a successful replication of the original. Before discussing in detail the replication issues, we briefly evaluate the model.

### 3.2. Model evaluation

Over the course of replicating the model by Maes et al. (2020), we have developed an in-depth understanding of its strengths and weaknesses. The model successfully learns to replay higher-order sequences through biologically plausible ingredients. It captures both elements specific duration and order, while bridging intrinsic timescales of neurons and synapses (few milliseconds) to behavioral timescales (hundreds of milliseconds). The decomposition of the architecture into one component that discretizes the time, and another that learns the spatial information, permits mapping different types of sequence elements to different time intervals. This flexibility allows the model to learn diverse spatiotemporal sequences.

Despite these promising features, the model poses a number of limitations and some of its mechanisms are hard to reconcile with biology, such as the stereotypical sequential activity of the clock. Although the network uses unsupervised learning rules to exhibit the desired dynamics, training the clock requires alternating excitatory and inhibitory inputs provided in a sequential manner to specific sets of neurons. It could therefore be argued that the biologically problematic issue of supervised learning has been shifted from the network to the sophisticated setup of external input. To our knowledge, the biological structures that would support such a training signal have not yet been identified. Furthermore, learning sequences with arbitrary timings and durations is a challenging problem. The period of the clock is relatively small (~470 ms in our case), which restricts the duration of a sequence. Learning sequences with larger temporal scales (~seconds) necessitates extending the period of the clock, which is a costly operation requiring the network size to be scaled up, i.e, increasing the number of clusters. The activation of a single cluster in the clock is sustained for ~15 ms, which defines the duration of a discrete step within the temporal backbone of the clock. This step dictates the smallest time interval that can represent an element in a sequence. Learning sequences with finer temporal details requires either redesigning the learning rules or fine tuning network parameters. An additional issue with learning sequences using a clock network is that the presentation of a sequence must always be synchronized with the period of the clock. Therefore, the time at which the first cluster is active must be identified every round, which requires more than just local information.

The model of Maes et al. (2020) can learn sequences in parallel, but replays them separately only if an external inhibitory signal is selectively provided to all the read-out neurons that are not associated with the elements of the sequence to be replayed. These mechanisms explaining such an inhibitory signal are hard to explain biologically. In addition, if the sequences to be learned in parallel contain the same elements, a new read-out neuron is needed for each one of these elements, even though they constitute the same alphabetic token, such as *ABCBA* and *AABBC*.

### 3.3. Implementation errors

In this study, we identify a number of implementation errors in the original code provided by Maes et al. (2020), some of which are shortly described below. The authors apparently apply Forward Euler to solve for the weight change of excitatory plasticity, i.e., using 3 as derivative and multiplying it by step size *dt*. However, the LTD term depends on the discontinuous and non-differentiable spike train of the presynaptic neuron and therefore Euler methods are non-applicable. Consequently, the simulation step size *dt* acts as a proportionality factor. Decreasing *dt* from 0.1 to 0.01 ms, which is supposed to increase the simulation accuracy, leads to vanishingly small long-term depression.

Confusion of 0- and 1-based indexing in the code also leads to a number of errors. For example, an excitatory neuron is partially treated as an inhibitory neuron, or clusters in the RNN are stimulated for 1 ms less than described in the text. Furthermore, some mistakes have been made in the computation of synaptic conductance. Inhibitory input is partially added to excitatory input, occasionally resulting in negative conductance values. The conductance calculation is further flawed by using double buffers only for a subset of state variables, leading to occasional erroneous usage of already modified synaptic weight values.

### 3.4. Parameter issues

The replication was hampered by inconsistent parameter definitions across text, tables and code. Some parameters are only mentioned in the text but not in the summarizing tables. This reduces the effectiveness of the tables as a resource for finding errors in a replication under development—any mistake in transferring the value of a parameter not mentioned in the tables will take much longer to find. Other parameters appeared only in the tables but not in the text. In this case, their role had to be conjectured from the source code. Additionally, some parameters are stated in neither the text nor the tables, for example, the synaptic delays. Also, no initial values such as the membrane potential or its low-pass filtered versions are specified. Furthermore, the code resets all state variables, except the synaptic weights, to their initial values every 2 minutes with no justification. After ensuring that this has no particular impact on the network dynamics, we refrain from resetting the values in our simulation. A table containing all parameters that have inconsistent values, are missing, or are only mentioned once in the paper, can be found in the Supplementary materials. Note that in the case of inconsistency between tables, text and code, we always take the source code as a final authority.

### 3.5. Absent and inconsistent information

Absent or inconsistent information hindered the replication beyond the issue of parameter specification. Examples of absent information include relevant details of the learning protocol or the equation defining the synaptic normalization procedure. With regards to inconsistent information, the low-pass filtering of spikes is defined differently in the text (Equation 12 in the original study) than in the code, resulting in substantially different synaptic weight developments before the issue is identified. In all cases, we are able to reconstruct the modelers' intentions by careful examination and instrumentation of the source code, which underlines the importance of providing this with every published numerical study.

### 3.6. Physical units

Most programming languages and tools do not take physical units into account. If using such tools, manual unit consistency checking of the equations is advisable to ensure the physical plausibility of all model components. We find unit mismatches in Equations (8), (9), and (14) of Maes et al. (2020). The kernel function (see Supplementary material), which enters the synaptic conductance through convolution, has a unit of ms^−1^ in Equation (8) of Maes et al. (2020). This kernel, however, must be unitless. To compensate, we introduce a constant *c*_K_ = 1 ms into the kernel function. Equations (9) and (14) of Maes et al. (2020) define excitatory and inhibitory plasticity, respectively. In Equation (9), the units of the learning rates *A*_LTD_ and *A*_LTP_ have been swapped, but even when corrected, the units of the left- and right-hand sides of the equation do not match. For this reason, we assign new units to the learning rates (see our 3). As for Equation (14), we modify the unit of the learning rate *A*_inh_ and introduce an additional constant α of unit ms^−1^ to the LTD term (see our 6).

### 3.7. Simulation and numerical techniques

There are advantages and disadvantages both to homebrewing code, as with the original study, or using purpose-built simulation tools, as with our replication. The source code for the model in Maes et al. (2020) is in Julia and MATLAB. These are general-purpose numeric computing platforms, consequently the researcher must develop all specific neuroscientific models and simulation algorithms from scratch, which presents a higher risk for implementation errors and poorly-suited numerics (Pauli et al., 2018). Examples from the current work include adding a variable intended to represent the conductance of an inhibitory synapse to a variable representing the excitatory input of a neuron, or using a Forward Euler solver for the neuron dynamics, resulting in membrane potentials that reach values of up to 17 mV. Domain-specific simulators such as NEST have key concepts (such as inhibition and excitation, or synaptic delays) integrated with their design, thus a lapse of attention by the developer will more likely result in a simulation time error, thereby flagging the problem and allowing it to be fixed. Similarly, the modeling language NESTML removes the burden of selecting and implementing appropriate numerical solvers. Additionally, it provides strict unit checking, substantially reducing the risk of implementing physically inconsistent equations as discussed above. Finally, as MATLAB is closed source and proprietary, it is less accessible than the available FOSS simulators and more prone to becoming non-executable legacy code, unless the code is regularly maintained (for an example, see Schulte To Brinke et al., 2022).

The major advantage of homebrewing code is the additional flexibility it gives the researcher. For example, synaptic normalization is not currently available in NEST as a built-in feature on the C++ level. Certainly it is faster to add features to a homebrewed solution than to an established simulator. However, a higher priority should be given to correct code than to rapid results—given the disadvantages of homebrewing discussed above, in the case that no available simulator has all the features one requires for a model, we would recommend selecting the simulator that is the best fit and working with the developers to fill the gap. If missing features cannot be incorporated into the simulator in a reasonable amount of time, the next best approach may well be a hybrid method, in which the simulator is supplemented with additional homebrewed features. Indeed, our implementation applies the synaptic normalization on the Python level, which is less performant than a C++ implementation would be, but still allows us to take full advantage of NEST's built-in models, algorithms and safety features such as type checking. Note that the implementation errors, physically inconsistent expressions and unsuitable numerics we find in the original code did not occur in the “missing feature” of synaptic normalization, but in the core simulator features such as solving neuronal and synaptic dynamics. Thus, many of these issues could have been avoided by using such a hybrid approach. We therefore conclude that resorting to a purely homebrewed framework should be a matter of last resort.

## 4. Methods

The model is described in detail in Maes et al. (2020). However, given the differences between the description of the model and its implementation (see Section 3), this chapter presents all important mechanisms and key components, which are essential for comprehension, replication, and validation. In addition, a detailed model table and all parameter values can be found in the Supplementary material. Furthermore, we make adjustments to physical units and added constants to resolve unit mismatches in some of the equations.

### 4.1. Network architecture

The model consists of two subnetworks: a recurrent network that learns the temporal pattern and a read-out layer responsible for learning the spatial patterns. The recurrent network (RNN) contains *N*^I^ inhibitory neurons (I) and *N*^E^ excitatory neurons (E). The excitatory neurons are divided uniformly into *N*^C^ clusters, each receiving a particular external input during training (see Section 4.4). A connection is created with probability *p* for each possible pair of neurons in the RNN, excluding autapses and multapses. Connections from excitatory to excitatory neurons (EE) and from inhibitory to excitatory neurons (EI) are plastic, all other connections are static. Plastic synapses have a lower bound *W*_min_ and an upper bound *W*_max_. The weights are initialized such that the network activity is in the E-I balanced state (Brunel, 2000).

The read-out layer contains *N*^R^ excitatory read-out neurons (R), each representing an element of the spatial dimension. There are no connections between the R neurons. Plastic all-to-all connections link excitatory neurons of the RNN to the read-out neurons (RE). Each read-out neuron is connected to an excitatory supervisor neuron (S) and an inhibitory interneuron (H).

### 4.2. Neuron and synapse models

**Excitatory neuron model**. The E, R, and S neurons are implemented as an adaptive exponential integrate-and-fire model (Brette and Gerstner, 2005):


(1) $$\begin{array}{l} \frac{dV^{\text{exc}}}{dt}=\frac{1}{\tau_{\text{E}}}\left(E_{\text{L}}^{\text{E}}-V^{\text{exc}}+\Delta_{\text{T}}^{\text{E}}\exp\left(\frac{V^{\text{exc}}-V_{\text{T}}^{\text{E}}}{\Delta_{\text{T}}^{\text{E}}}\right)\right)\\ \;-\gamma_{\text{x}}\frac{a^{\text{E}}}{C}+Q_{\text{x}},\;x\in \left\{\text{E,R,S}\right\} \end{array}$$

where τ_E_ is the membrane time constant, $E_{\text{L}}^{\text{E}}$ is the reversal potential, $V_{\text{T}}^{\text{E}}$ is an adaptive threshold, $\Delta_{\text{T}}^{\text{E}}$ is the slope of the exponential, *a*^E^ is the adaption current, and *Q*_*x*_ is the current received by the neuron. Only E neurons implement synaptic adaptation (γ_E_ = 1,γ_S_ = 0, γ_R_ = 0). More details about the adaptive threshold $V_{\text{T}}^{\text{E}}$, the adaption current *a*^E^ and the received current *Q*_*x*_ can be found in the Supplementary material.

**Inhibitory neuron model**. The I and H neurons are implemented as a leaky integrate-and-fire model:


(2) $$\begin{array}{l} \frac{dV^{\text{inh}}}{dt}=\frac{E_{\text{L}}^{\text{I}}-V^{\text{inh}}}{\tau_{\text{I}}}+Q_{x},\;x\in \left\{\text{I,H}\right\} \end{array}$$

where τ_I_ is the membrane time constant, $E_{\text{L}}^{\text{I}}$ is the reversal potential and *Q*_*x*_ is the current received by the neuron.

### 4.3. Plasticity

**Excitatory plasticity**. The synaptic weights from excitatory to excitatory neurons (EE) and from excitatory to read-out neurons (RE) evolve according to a voltage-based STDP rule (Clopath et al., 2010):


(3) $$\begin{array}{l} \begin{array}{c} \frac{dW_{ij}^{\text{EE, RE}}}{dt}=-A_{\text{LTD}}s_{j}\left(t-d\right)R\left(u_{i}\left(t\right)-\Theta_{\text{LTD}}\right)\\ \;+A_{\text{LTP}}x_{j}\left(t-d\right)R\left(V_{\text{i}}\left(t\right)-\Theta_{\text{LTP}}\right)\\ R\left(v_{\text{i}}\left(t\right)-\Theta_{\text{LTD}}\right), \end{array} \end{array}$$

where *A*_LTD_ (*A*_LTP_) is the learning rate and Θ_LTD_ (Θ_LTP_) the voltage threshold for depression (potentiation), *R*(*x*) is a linear-rectifying function, *d* is the synaptic delay, and *V*_*i*_ is the postsynaptic membrane potential. The traces *u*_*i*_ and *v*_*i*_ are low-pass filtered versions of *V*_*i*_ with the corresponding time constants τ_u_ and τ_v_


(4) $$\begin{array}{l} \frac{du_{i}}{dt}=\frac{1}{\tau_{\text{u}}}\left(V_{i}-u_{i}\right) \end{array}$$

and analog for *v*_*i*_,τ_v_. The presynaptic spike train is $s_{j}\left(t\right)=\underset{k}{\sum }\delta \left(t-t_{j,k}\right)$, where δ is the Dirac delta function, and *t*_*j, k*_ is the *k*th spike time of neuron *j*. The potentiation term of 3 depends on the low-pass filtered version *x*_*j*_ of the spike train *s*_*j*_


(5) $$\begin{array}{l} \frac{dx_{j}}{dt}=\alpha s_{j}-\frac{x_{j}}{\tau_{x}}, \end{array}$$

with different values of time constant τ_*x*_ for synapses within the excitatory recurrent network (τ_xEE_) and synapses connecting to the read-out neurons (τ_xRE_). The parameter α is introduced to correct for unit mismatch. In contrast to Maes et al. (2020), we omit the weight dependent potentiation in the voltage-based STDP rule applied to the RE connections.

**Inhibitory Plasticity**. The synaptic weights from inhibitory neurons to excitatory neurons evolve according to the plasticity rule proposed by Vogels et al. (2011), where the synaptic change is driven by either pre- or postsynaptic spikes:


(6) $$\begin{array}{l} \begin{array}{cc} \frac{dW_{ij}^{\text{EI}}}{dt} & =A_{\text{inh}}\left(y_{i}^{\text{E}}\left(t\right)-2\alpha r_{0}\tau_{y}\right)s_{j}^{\text{I}}\left(t-d\right)+A_{\text{inh}}y_{j}^{\text{I}}\left(t\right)s_{i}^{\text{E}}\left(t\right). \end{array} \end{array}$$

Here, *A*_inh_ is the learning rate of inhibitory plasticity, *r*_0_ is the target firing rate, τ_*y*_ is the time constant, $s_{i}^{\text{E}}$ and $s_{j}^{\text{I}}$ are spike trains of the excitatory postsynaptic neuron *i* and the inhibitory presynaptic neuron *j*, respectively, *d* is the synaptic delay, and $y_{i}^{\text{E}}$ and $y_{j}^{\text{I}}$ are the corresponding low-pass filtered versions, which can be derived with the time constant τ_*y*_ as in 5. The parameter α is introduced to correct for unit mismatch (see Section 3).

**Synaptic Normalization**. The connections from excitatory to excitatory neurons within the recurrent network (EE) exhibit synaptic normalization. This is achieved by keeping the sum of all incoming weights to a neuron *i* constant at regular intervals τ_norm_ by applying


(7) $$\begin{array}{l} W_{ij}\leftarrow W_{ij}-\frac{\left(\overset{N^{\text{E}}}{\underset{k=1}{\sum }}W_{ik}\right)-\text{K}}{l},\;\text{K}=\overset{N^{\text{E}}}{\underset{k=1}{\sum }}W_{ik}^{0}, \end{array}$$

where *l* is the number of incoming connections.

### 4.4. Learning protocol

The learning undergoes two stages. First, the RNN learns the sequential dynamics and then the connections from the RNN to the read-out layer are learned to represent the respective sequence.

**Recurrent network**. The learning of the internal clock, which exhibits sequential dynamics, is divided into two learning phases. In the first phase, the clusters are stimulated one by one with external excitatory input from a Poisson process with rate $r_{\text{exc}1}^{\text{E}}$: starting at cluster 1, each cluster is stimulated in ascending order for 9 ms. Gaps of 6 ms each separate the excitation periods of two adjacent clusters. When the last cluster (here, the 30th), has been excited, the stimulation starts again at the first cluster. Once a cluster is no longer receiving excitatory input, each of its neurons immediately receives inhibitory external input with rate $r_{\text{inh}}^{\text{E}}$. Throughout the first phase, inhibitory neurons are stimulated at a constant rate of $r_{\text{exc}}^{\text{I}}$. The sequential routine is performed around 8, 000 times, resulting in 1 hour of biological time.

The second learning phase of the clock is carried out for one additional hour, during which all excitatory neurons receive ongoing external excitatory input with rate $r_{\text{exc}2}^{\text{E}}$. Input to inhibitory neurons remains the same as in the first learning phase. Both Poisson generators stay active over the whole time interval.

After the second phase, the connectivity stabilizes. The sequential dynamics get encoded in the recurrent network and can be activated by external spontaneous input ($r_{\text{exc}}^{\text{I}}$ and $r_{\text{exc2}}^{\text{E}}$). Nevertheless, the weights of all connections in the clock are frozen after the learning of the sequential dynamics has succeeded.

**Read-out Layer**. During learning of the read-out synapses, external input drives both supervisor neurons as well as interneurons. Each element of the sequence is assigned to one of the read-out neurons and its supervisor neuron. Thus, an element of the sequence is learned by producing and sending a spike train with rate $r_{\text{exc}}^{\text{S}}$ to the associated S neuron for a certain time interval. Meanwhile all other supervisor neurons receive a baseline input with rate $r_{\text{base}}^{\text{S}}$.

Before the sequence is learned, the RNN must first exhibit sequential dynamics, which is ensured by stimulating the network using spontaneous input ($r_{\text{exc}}^{\text{I}}$ and $r_{\text{exc2}}^{\text{E}}$). After the sequential dynamics have stabilized, the sequence is presented to the network by sequentially stimulating the associated supervisor neuron for 75 ms with rate $r_{\text{exc}}^{\text{S}}$ for each element. Throughout the learning, each interneuron receives a constant input with rate $r_{\text{exc}}^{\text{H}}$. The sequence is repeatedly shown to the network for 12 seconds, with each element always appearing at the same time relative to the activation of the first cluster. After training has been completed, supervisor neurons and interneurons no longer receive external input. Replay of the learned sequence can be triggered by external spontaneous input to the recurrent network alone.

The number of elements and their type has a strong influence on the learning of the read-out synapses, since it determines the number of read-out neurons and the intensity of the supervisor input.

### 4.5. Simulation details

The network simulations are performed in the neural simulator NEST 3.0 (Hahne et al., 2021). The inhibitory neurons are defined in the domain-specific language NESTML 5.0 (Babu et al., 2021), which generates the required C++ code for the dynamic loading into NEST.

## Data availability statement

Our PyNEST implementation is available on Zenodo at https://doi.org/10.5281/zenodo.7046137. The original source code used in Maes et al. (2020) can be found in a ModelDB repository at http://modeldb.yale.edu/257609.

## Author contributions

JO, YB, and AM designed the study and contributed to writing of manuscript. JO re-implemented the model and performed all simulations and analyzes. All authors contributed to the article and approved the submitted version.

## Funding

This work has received partial support from the Initiative and Networking Fund of the Helmholtz Association, the Helmholtz Portfolio Theme Supercomputing and Modeling for the Human Brain, and the Excellence Initiative of the German Federal and State Governments (G:(DE-82)EXS-SFneuroIC002). Open access publication was funded by the Deutsche Forschungsgemeinschaft (DFG, German Research Foundation; Grant 491111487).

## Conflict of interest

The authors declare that the research was conducted in the absence of any commercial or financial relationships that could be construed as a potential conflict of interest.

## Publisher's note

All claims expressed in this article are solely those of the authors and do not necessarily represent those of their affiliated organizations, or those of the publisher, the editors and the reviewers. Any product that may be evaluated in this article, or claim that may be made by its manufacturer, is not guaranteed or endorsed by the publisher.
