# Supplementary material for: Learning and replaying spatiotemporal sequences: A replication study
Source: Front Integr Neurosci. 2022 Oct 14;16:974177. doi: 10.3389/fnint.2022.974177 (PMC9614051; doi:10.3389/fnint.2022.974177)
Supplement: Supplementary file 1 [file Data_Sheet_1.pdf]

Supplementary Material

1 MODEL TABLES

**Summary**	
**Populations**	excitatory population $\mathcal{E}$, inhibitory population $\mathcal{I}$, read-out population $\mathcal{R}$, supervisor population $\mathcal{S}$, interneuron population $\mathcal{H}$, and external spike-train generators $\mathcal{X}_{\mathrm{E}}$,$\mathcal{X}_{\mathrm{I}}$,x_{I},$\mathcal{X}_{\mathrm{S}}$,x_{H}.
**Connectivity**	$\mathcal{E} \cup \mathcal{I} \to \mathcal{E} \cup \mathcal{I}$: pairwise with probability p; $\mathcal{E} \to \mathcal{R}$: all-to-all; $\mathcal{S} \to \mathcal{R}$: one-to-one; $\mathcal{H} \to \mathcal{R}$: one-to-one; $\mathcal{R} \to \mathcal{H}$: one-to-one; see below for the external inputs
**Neuron model**	adaptive exponential integrate-and-fire model (AEIF, AdEx) and leaky integrate-and-fire model (LIF)
**Synapse model**	conductance-based, excitatory plasticity using voltage-based STDP (Clopath et al., 2010), and inhibitory plasticity by symmetric STDP with constant depression (Vogels et al., 2011)
**Input**	Poissonian spike trains

**Populations**		
**Name**	**Elements**	**Size**
$\mathcal{E} = \cup_{k=0}^{N^{\mathrm{C}}} \mathcal{C}_k$	AEIF	N^{E}
$\mathcal{C}_k$	AEIF	$N^{\mathrm{E}}/N^{\mathrm{C}}$
$\mathcal{I}$	I&F	N^{I}
$\mathcal{R}$	AEIF	N^{R}
$\mathcal{S}$	AEIF	N^{S}
$\mathcal{H}$	I&F	N^{H}
$\mathcal{X}_{\mathrm{E}}$,$\mathcal{X}_{\mathrm{I}}$,x_{I},$\mathcal{X}_{\mathrm{S}}$,x_{H}	spike-train generators (Poisson)	N^{C}, N^{C}, 1, N^{S}, 1

**Connectivity**		
**Source**	**Target**	**Pattern**
$\mathcal{E}$	$\mathcal{E} \cup \mathcal{I}$	For each possible pair, a connection is created with connection probability p; delays $d_{ij} = d$; plastic weights W_{ij} ($\forall i \in \mathcal{E}$, $\forall j \in \mathcal{E}$); static weights W_{ij} ($\forall i \in \mathcal{I}$, $\forall j \in \mathcal{E}$)
$\mathcal{I}$	$\mathcal{E} \cup \mathcal{I}$	For each possible pair, a connection is created with connection probability p; delays $d_{ij} = d$; plastic weights W_{ij} ($\forall i \in \mathcal{E}$, $\forall j \in \mathcal{I}$); static weights W_{ij} ($\forall i \in \mathcal{I}$, $\forall j \in \mathcal{I}$)
$\mathcal{E}$	$\mathcal{R}$	all-to-all; delays $d_{ij} = d$; plastic weights W_{ij} ($\forall i \in \mathcal{R}$, $\forall j \in \mathcal{E}$)
$\mathcal{H}$	$\mathcal{R}$	one-to-one; delays $d_{ij} = d$; static weights $W_{ij} = W^{\mathrm{RH}}$ ($\forall i \in \mathcal{R}$, $\forall j \in \mathcal{H}$)
$\mathcal{S}$	$\mathcal{R}$	one-to-one; delays $d_{ij} = d$; static weights $W_{ij} = W^{\mathrm{RS}}$ ($\forall i \in \mathcal{R}$, $\forall j \in \mathcal{S}$)
$\mathcal{R}$	$\mathcal{H}$	one-to-one; delays $d_{ij} = d$; static weights $W_{ij} = W^{\mathrm{HR}}$ ($\forall i \in \mathcal{H}$, $\forall j \in \mathcal{R}$)
$\mathcal{X}_{\mathrm{E},k}$	$\mathcal{C}_k$	one-to-all; delays $d_{ij} = d$; static weights $W_{ij} = W_{\mathrm{exc}}^{\mathrm{EX}}$ ($\forall i \in \mathcal{C}_k$, $\forall j \in \mathcal{X}_{\mathrm{E},k}$)
$\mathcal{X}_{\mathrm{I},k}$	$\mathcal{C}_k$	one-to-all; delays $d_{ij} = d$; static weights $W_{ij} = W_{\mathrm{inh}}^{\mathrm{EX}}$ ($\forall i \in \mathcal{C}_k$, $\forall j \in \mathcal{X}_{\mathrm{I},k}$)
x_{I}	$\mathcal{I}$	one-to-all; delays $d_{ij} = d$; static weights $W_{ij} = W_{\mathrm{exc}}^{\mathrm{IX}}$ ($\forall i \in \mathcal{I}$, $j \in x_{\mathrm{I}}$)
$\mathcal{X}_{\mathrm{S}}$	$\mathcal{S}$	one-to-one; delays $d_{ij} = d$; static weights $W_{ij} = W_{\mathrm{exc}}^{\mathrm{SX}}$ ($\forall i \in \mathcal{S}$, $\forall j \in \mathcal{X}_{\mathrm{S}}$)
x_{H}	$\mathcal{H}$	one-to-all; delays $d_{ij} = d$; static weights $W_{ij} = W_{\mathrm{exc}}^{\mathrm{HX}}$ ($\forall i \in \mathcal{H}$, $\forall j \in \mathcal{X}_{\mathrm{H}}$)
all	all	no self-connections (“autapses”), no multiple connections between the same pair (“multapses”)

Table S1. Description of the network model (continued).

<table>
<tr><th colspan="2">Excitatory Neuron</th></tr>
<tr><td>Type</td><td>adaptive exponential integrate-and-fire model (AEIF, AdEx)</td></tr>
<tr><td>Description</td><td>dynamics of membrane potential $V_i^{\text{exc}}(t)$ and spiking activity $s_i(t)$ of excitatory (E), read-out (R), and supervisor (S) neuron with index i:
• supra-threshold dynamics:
• emission of kth ($k = 1, 2, \ldots$) spike of neuron i at time t_i^k if
$$V_i^{\text{exc}}\left(t_i^k\right) \geq V_{\text{peak}}$$
• spike train: $s_i(t) = \sum_k \delta(t - t_i^k)$
• clamping of membrane potential: $V_i^{\text{exc}}(t) = V_{\text{r}}$ for $t \in (t_i^k, t_i^k + \tau]$, where $\tau = \tau_{\text{abs}}$ for E neurons and $\tau = \tau_{\text{absR}} = \tau_{\text{absS}}$ for R or S neurons
• spike initiation threshold reset: $V_{\text{T},i}^{\text{E}}(t_i^k) = A_{\text{T}} + V_{\text{T}}$
with reset potential V_{r}, refractory period τ, spike threshold V_{T}^{E}, adaptive threshold increment A_{T}, resting threshold V_{T}
• sub-threshold dynamics:
• membrane potential:
$$\frac{dV_i^{\text{exc}}}{dt} = \frac{1}{\tau_{\text{E}}}\left(E_{\text{L}}^{\text{E}} - V_i^{\text{exc}} + \Delta_{\text{T}}^{\text{E}} \exp\left(\frac{V_i^{\text{exc}} - V_{\text{T},i}^{\text{E}}}{\Delta_{\text{T}}^{\text{E}}}\right)\right) - \gamma_x \frac{a_i^{\text{E}}}{C} + Q_x, \quad \text{with } x \in \{\text{E,R,S}\} \qquad \text{(S1)}$$
where τ_{E} is the excitatory membrane time constant, E_{L}^{E} is the excitatory reversal potential, V_{T}^{E} is an adaptive threshold and $\Delta_{\text{T}}^{\text{E}}$ is the slope of the exponential, and Q_x is the conductance current. Only E neurons implement synaptic adaptation ($\gamma_{\text{E}} = 1$,$\gamma_{\text{S}} = 0$, $\gamma_{\text{R}} = 0$).
• spike initiation threshold:
$$\tau_{\text{T}} \frac{dV_{\text{T},i}^{\text{E}}}{dt} = V_{\text{T}} - V_{\text{T},i}^{\text{E}}, \qquad \text{(S2)}$$
where τ_{T} is the spike initiation threshold time constant and V_{T} is the resting threshold
• adaptation current:
$$\tau_{\text{a}} \frac{da_i^{\text{E}}}{dt} = -a_i^{\text{E}}, \qquad \text{(S3)}$$
with τ_{a} is the time constant. The adaptation current a^{E} is increased with a constant β when the neuron i emits a spike.</td></tr>
</table>

Table S2. Description of the network model (continued).

Inhibitory neuron	
**Type**	leaky integrate-and-fire model (LIF)
**Description**	dynamics of membrane potential $V_i^{\text{inh}}(t)$ and spiking activity $s_i(t)$ of inhibitory neuron (I) and interneuron (H) with index i:<br>• membrane potential: $$\frac{dV_i^{\text{inh}}}{dt} = \frac{E_{\text{L}}^{\text{I}} - V_i^{\text{inh}}}{\tau_{\text{I}}} + Q_x \; x \in \{\text{I,H}\}, \quad \text{(S4)}$$ where τ_{I} is the inhibitory membrane time constant, E_{L}^{I} is the inhibitory reversal potential, and Q_x is the conductance current<br>• emission of a spike: $V_i^{\text{inh}}\left(t_i^k\right) \geq V^{\text{T}}$<br>• spike train: $s_i(t) = \sum_k \delta(t - t_i^k)$<br>• clamping of membrane potential: $V_i^{\text{inh}}(t) = V_{\text{r}}$ for $t \in (t_i^k, t_i^k + \tau]$, where $\tau = \tau_{\text{abs}}$ for I neurons and $\tau = \tau_{\text{absH}}$ for H neurons

Table S3. Description of the network model (continued).

Synapses	
**Type**	conductance-based synapses
**Description**	• synaptic input of a neuron i corresponding to E, R, S, I, H neurons: $$Q_{\mathrm{E},i}(t) = g_{\mathrm{exc}}^{\mathrm{EX}} \frac{E^{\mathrm{E}} - V_i^{\mathrm{exc}}}{C} + g_{\mathrm{inh}}^{\mathrm{EX}} \frac{E^{\mathrm{I}} - V_i^{\mathrm{exc}}}{C} + g^{\mathrm{EE}} \frac{E^{\mathrm{E}} - V_i^{\mathrm{exc}}}{C} + g^{\mathrm{EI}} \frac{E^{\mathrm{I}} - V_i^{\mathrm{exc}}}{C}$$ $$Q_{\mathrm{R},i}(t) = g^{\mathrm{RE}} \frac{E^{\mathrm{E}} - V_i^{\mathrm{exc}}}{C} + g^{\mathrm{RS}} \frac{E^{\mathrm{E}} - V_i^{\mathrm{exc}}}{C} + g^{\mathrm{RH}} \frac{E^{\mathrm{I}} - V_i^{\mathrm{exc}}}{C}$$ $$Q_{\mathrm{S},i}(t) = g_{\mathrm{exc}}^{\mathrm{SX}} \frac{E^{\mathrm{E}} - V_i^{\mathrm{exc}}}{C}$$ $$Q_{\mathrm{I},i}(t) = g_{\mathrm{exc}}^{\mathrm{IX}} \frac{E^{\mathrm{E}} - V_i^{\mathrm{inh}}}{C} + g^{\mathrm{IE}} \frac{E^{\mathrm{E}} - V_i^{\mathrm{inh}}}{C} + g^{\mathrm{II}} \frac{E^{\mathrm{I}} - V_i^{\mathrm{inh}}}{C}$$ $$Q_{\mathrm{H},i}(t) = g_{\mathrm{exc}}^{\mathrm{HX}} \frac{E^{\mathrm{E}} - V_i^{\mathrm{inh}}}{C} + g^{\mathrm{HR}} \frac{E^{\mathrm{E}} - V_i^{\mathrm{inh}}}{C}$$ where g^{xy} is the synaptic conductance where x denotes the target and y denotes the source, $g_{\mathrm{exc}}^{x\mathrm{X}}$ is the synaptic conductance through excitatory external input, $g_{\mathrm{inh}}^{x\mathrm{X}}$ is the synaptic conductance through inhibitory external input, E^{E} is the excitatory reversal potential, and E^{I} is the inhibitory reversal potential,<br>• synaptic conductance: $$g_i^{xy}(t) = K^x(t) * \left(\sum_{j \in y} W_{ij}^{xy} \cdot s_j(t-d) \right) \quad \text{(S5)}$$ $$g_{\mathrm{exc},i}^{x\mathrm{X}}(t) = K^x(t) * \left(\sum_{j \in \mathrm{X}} W_{\mathrm{exc},ij}^{x\mathrm{X}} \cdot s_j(t-d) \right) \quad \text{(S6)}$$ $$g_{\mathrm{inh},i}^{x\mathrm{X}}(t) = K^x(t) * \left(\sum_{j \in \mathrm{X}} W_{\mathrm{inh},ij}^{x\mathrm{X}} \cdot s_j(t-d) \right) \quad \text{(S7)}$$ • synaptic kernel: $$K^x(t) = c_{\mathrm{K}} \left(\frac{\exp^{-t/\tau_{\mathrm{d}}^x} - \exp^{-t/\tau_{\mathrm{r}}^x}}{\tau_{\mathrm{d}}^x - \tau_{\mathrm{r}}^x} \right) \quad \text{(S8)}$$ where τ_{d}^x is the decay time and τ_{r}^x is the rise time. In contrast to (Maes et al., 2020), we introduce the constant c_{K} with value $1\ \mathrm{ms}$ to keep the kernel unitless.

Table S4. Description of the network model (continued).

Plasticity	
**Type**	excitatory plasticity using voltage-based STDP (Clopath et al., 2010) and inhibitory plasticity by symmetric STDP with constant depression (Vogels et al., 2011)
**Description**	• excitatory plasticity ($\forall i \in \mathcal{E} \cup \mathcal{R}, \forall j \in \mathcal{E}$)<br>• dynamics of the synaptic weights $W_{ij}(t)$:<br>$$\frac{dW^{\text{EE, RE}}_{\text{ij}}}{dt} = -A_{\text{LTD}}\, s_j(t-d)\, R(u_{\text{i}}(t) - \Theta_{\text{LTD}}) + A_{\text{LTP}}\, x_j(t-d)\, R(V_i(t) - \Theta_{\text{LTP}})\, R(v_i(t) - \Theta_{\text{LTD}}), \quad \text{(S9)}$$<br>where A_{LTD} and A_{LTP} are the learning rates for depression and potentiation, d is the synaptic delay, Θ_{LTD} and Θ_{LTP} are the voltage thresholds for depression and potentiation, respectively, and $R(x)$ is a linear-rectifying function.<br>• low pass filtered membrane potentials $V_i(t)$<br>$$\frac{d\overline{V_i}}{dt} = \frac{1}{\tau}(V_i - \overline{V_i}) \text{ with } (\overline{V_i}, \tau) \in \{(u_i, \tau_{\text{u}}), (v_i, \tau_{\text{v}})\}, \quad \text{(S10)}$$<br>where τ_{u} and τ_{v} are the time constants of the filtered membrane potentials u_i and v_i, respectively.<br>• low-pass filtered spike train $x_j(t)$<br>$$\frac{dx_j}{dt} = \alpha s_j - \frac{x_j}{\tau_x} \text{ with } \tau_x \in \{\tau_{\text{xEE}}, \tau_{\text{xRE}}\}, \quad \text{(S11)}$$<br>where τ_x is the time constant and equals τ_{xEE} for EE connections and τ_{xRE} for RE connections. The parameter α is introduced to correct for unit mismatch.<br>• inhibitory plasticity ($\forall i \in \mathcal{E}, j \in \mathcal{I}$)<br>• dynamics of the synaptic weights $W_{ij}(t)$:<br>$$\frac{dW^{\text{EI}}_{ij}}{dt} = A_{\text{inh}} \left(y^{\text{E}}_i(t) - 2\alpha r_0 \tau_y\right) s^{\text{I}}_j(t-d) + A_{\text{inh}}\, y^{\text{I}}_j(t)\, s^{\text{E}}_i(t), \quad \text{(S12)}$$<br>where A_{inh} is the learning rate of inhibitory plasticity, r_0 is the target firing rate, τ_y is the time constant, d is the synaptic delay, s^{E}_i and s^{I}_j are the spike trains of postsynaptic neuron i and presynaptic neuron j, and y^{E}_i and y^{I}_j are the corresponding low-pass filtered versions, which can be derived with the time constant τ_y as in Equation S10. The parameter α is introduced to correct for unit mismatch.<br>• synaptic normalization ($\forall i \in \mathcal{E}, \forall j \in \mathcal{E}$)<br>• update of the synaptic weights $W_{ij}(t)$ at regular intervals τ_{norm}:<br>$$W^{\text{EE}}_{ij} \leftarrow W^{\text{EE}}_{ij} - \frac{\left(\sum_{k=1}^{N^{\text{E}}} W^{\text{EE}}_{ik}\right) - \text{K}}{l}, \ \text{K} = \sum_{k=1}^{N^{\text{E}}} W^{\text{EE,0}}_{ik}, \quad \text{(S13)}$$<br>where l is the number of incoming connections.<br>• all plastic synapses have a lower bound W_{min} and upper bound W_{max}.

Table S5. Description of the network model (continued).

Stimulus	
**Type**	Poissonian spike-train generators
**Description**	See the training protocol in the main text of how the rates of the different external sources are adjusted during the different training phases.

Initial conditions
• membrane potentials of excitatory neurons: $V_i^{\text{exc}}(0) = V_0$<br>• membrane potentials of inhibitory neurons: $V_i^{\text{inh}}(0) = V_0$<br>• excitatory synaptic weights (EE) $W_{ij}(0) = W_0^{\text{EE}}$ $(\forall i \in \mathcal{E}, \forall j \in \mathcal{E})$<br>• inhibitory synaptic weights (EI) $W_{ij}(0) = W_0^{\text{EI}}$ $(\forall i \in \mathcal{E}, \forall j \in \mathcal{I})$<br>• excitatory synaptic weights to read-out (RE) $W_{ij}(0) = W_0^{\text{RE}}$ $(\forall i \in \mathcal{R}, \forall j \in \mathcal{E})$<br>• spike initiation threshold $V_{\text{T}}^{\text{E}}(0) = V_{\text{T,0}}^{\text{E}}$<br>• adaptation current $a_i^{\text{E}}(0) = 0\,\text{pA}$<br>• low-pass filtered membrane potentials $\bar{V}_i(0) = 0\,\text{mV}$<br>• low-pass filtered spike trains $x_i(0) = 0\,\text{ms}^{-1}$

Table S6. Description of the network model.

2 PARAMETER TABLES

Network		
**Name**	**Value**	**Description**
N^{E}	2400	Number of excitatory neurons (E)
N^{I}	600	Number of inhibitory neurons (I)
N^{C}	30	Number of excitatory clusters
N^{R}	3	Number of read-out neurons (R)
N^{S}	3	Number of supervisor neurons (S)
N^{H}	3	Number of interneurons (I)
p	0.2	Connection probability in recurrent network
**Simulation and initial conditions**		
dt	$0.1\ \mathrm{ms}$	Resolution time
W_0^{EE}	$2.83\ \mathrm{pF}$	Initial excitatory synaptic strength from E to E neurons
W_0^{EI}	$62.87\ \mathrm{pF}$	Initial inhibitory synaptic strength from I to E neurons
W_0^{RE}	$0\ \mathrm{pF}$	Initial excitatory synaptic strength from E to R neurons
W^{IE}	$1.96\ \mathrm{pF}$	Excitatory synaptic strength from E to I neurons
W^{II}	$20.91\ \mathrm{pF}$	Inhibitory synaptic strength from I to I neurons
W^{RS}	$200\ \mathrm{pF}$	Excitatory synaptic strength from S to R neurons
W^{RH}	$200\ \mathrm{pF}$	Inhibitory synaptic strength from H to R neurons
W^{HR}	$200\ \mathrm{pF}$	Excitatory synaptic strength from R to H neurons
V_0	$-60\ \mathrm{mV}$	Initial membrane potential
u_0, v_0	$0\ \mathrm{mV}$	Initial low-pass filtered membrane potential
a_0^{E}	$0\ \mathrm{pA}$	Initial adaption current
$V_{\mathrm{T},0}^{\mathrm{E}}$	$-52\ \mathrm{mV}$	Initial adaptive threshold

Table S7. Initialization of network.

Membrane and synaptic dynamics		
**Name**	**Value**	**Description**
τ_{E}	$20\ \mathrm{ms}$	E membrane potential time constant
τ_{I}	$20\ \mathrm{ms}$	I membrane potential time constant
τ_{abs}	$5\ \mathrm{ms}$	Refractory period of E and I neurons
τ_{absR}	$1\ \mathrm{ms}$	Refractory period of R neurons
τ_{absS}	$1\ \mathrm{ms}$	Refractory period of S neurons
τ_{absH}	$1\ \mathrm{ms}$	Refractory period of H neurons
τ_{T}	$30\ \mathrm{ms}$	Adaptive threshold time constant
E^{E}	$0\ \mathrm{mV}$	Excitatory reversal potential
E^{I}	$-75\ \mathrm{mV}$	Inhibitory reversal potential
$E_{\mathrm{L}}^{\mathrm{E}}$	$-70\ \mathrm{mV}$	Excitatory resting potential
$E_{\mathrm{L}}^{\mathrm{I}}$	$-62\ \mathrm{mV}$	Inhibitory resting potential
V_{r}	$-60\ \mathrm{mV}$	Reset potential of all neurons
V_{peak}	$20\ \mathrm{mV}$	Spike threshold of excitatory neurons
V_{T}	$-52\ \mathrm{mV}$	Membrane potential threshold
A_{T}	$10\ \mathrm{mV}$	Adaptive threshold increase constant
C	$300\ \mathrm{pF}$	Capacitance
$\Delta_{\mathrm{T}}^{\mathrm{E}}$	$2\ \mathrm{mV}$	Exponential slope
τ_{a}	$100\ \mathrm{ms}$	Adaption current time constant
β	$1000\ \mathrm{pA}$	Adaption current increase constant in RNN
γ_{x}	$\gamma_{\mathrm{E}} = 1$, $\gamma_{x\in\{\mathrm{R,S}\}=0}$	Selector if adaption current is present for neuron $x \in \{\mathrm{E,R,S}\}$
$\tau_{\mathrm{d}}^{\mathrm{E}}$	$6\ \mathrm{ms}$	Excitatory decay constant
$\tau_{\mathrm{r}}^{\mathrm{E}}$	$1\ \mathrm{ms}$	Excitatory rise constant
$\tau_{\mathrm{d}}^{\mathrm{I}}$	$2\ \mathrm{ms}$	Inhibitory decay constant
$\tau_{\mathrm{r}}^{\mathrm{I}}$	$0.5\ \mathrm{ms}$	Inhibitory rise constant
c_{K}	$1\ \mathrm{ms}$	Compensation constant
d	$0.1\ \mathrm{ms}$	Synaptic delay
τ_{norm}	$450\ \mathrm{ms}$	Time interval between normalization steps

Table S8. Neuronal membrane and synaptic dynamics parameters.

Plasticity parameters		
**Name**	**Value**	**Description**
A_{LTD}	$0.00014\ \frac{\mathrm{pF}}{\mathrm{mV}}$	LTD amplitude of excitatory plasticity
A_{LTP}	$0.0008\ \frac{\mathrm{pF}}{\mathrm{mV}^2}$	LTP amplitude of excitatory plasticity
Θ_{LTD}	-70 mV	LTD threshold of excitatory plasticity
Θ_{LTP}	-49 mV	LTP threshold of excitatory plasticity
τ_{u}	10 ms	Time constant of low pass filtered postsynaptic membrane potential (LTD)
τ_{v}	7 ms	Time constant of low pass filtered postsynaptic membrane potential (LTP)
$d_{\mathrm{u,v}}$	0.1 ms	Delay for processing the low-pass filtered membrane potentials
τ_{xEE}	3.5 ms	Time constant of low pass filtered spike train in recurrent network for excitatory plasticity
τ_{xRE}	5 ms	Time constant of low pass filtered spike train for read-out synapses
α	$1\ \frac{1}{\mathrm{ms}}$	Compensation constant
A_{inh}	1 pF ms	Amplitude of inhibitory plasticity
r_0	3 Hz	Target firing rate for inhibitory plasticity
τ_y	20 ms	Time constant of low pass filtered spike train in recurrent network for inhibitory plasticity
$W_{\mathrm{min}}^{\mathrm{EE}}$	1.45 pF	Minimum E to E synaptic strength
$W_{\mathrm{max}}^{\mathrm{EE}}$	32.68 pF	Maximum E to E synaptic strength
$W_{\mathrm{min}}^{\mathrm{RE}}$	0 pF	Minimum E to R synaptic strength
$W_{\mathrm{max}}^{\mathrm{RE}}$	25 pF	Maximum E to R synaptic strength
$W_{\mathrm{min}}^{\mathrm{EI}}$	48.7 pF	Minimum I to E synaptic strength
$W_{\mathrm{max}}^{\mathrm{EI}}$	243 pF	Maximum I to E synaptic strength

Table S9. Plasticity parameters.

External input		
**Name**	**Value**	**Description**
$r^{\mathrm{E}}_{\mathrm{exc1}}$	$22.5\ \mathrm{k\ spks/sec}$	Rate of sequential external excitatory input to E neurons
$r^{\mathrm{E}}_{\mathrm{exc2}}$	$4.5\ \mathrm{k\ spks/sec}$	Rate of spontaneous external excitatory input to E neurons
$r^{\mathrm{E}}_{\mathrm{inh}}$	$4.5\ \mathrm{k\ spks/sec}$	Rate of sequential external inhibitory input to E neurons
$r^{\mathrm{I}}_{\mathrm{exc}}$	$2.25\ \mathrm{k\ spks/sec}$	Rate of external excitatory input to I neurons
$r^{\mathrm{S}}_{\mathrm{exc}}$	$10\ \mathrm{k\ spks/sec}$	Rate of external excitatory supervisor input to S neurons
$r^{\mathrm{S}}_{\mathrm{base}}$	$1\ \mathrm{k\ spks/sec}$	Baseline rate of external excitatory input to S neurons
$r^{\mathrm{H}}_{\mathrm{exc}}$	$1\ \mathrm{k\ spks/sec}$	Rate of external excitatory input to H neurons
$W^{\mathrm{EX}}_{\mathrm{exc}}$	$1.6\ \ \mathrm{pF}$	Excitatory external input strength to E neurons
$W^{\mathrm{EX}}_{\mathrm{inh}}$	$0.8\ \ \mathrm{pF}$	Inhibitory external input strength to E neurons
$W^{\mathrm{IX}}_{\mathrm{exc}}$	$1.52\ \ \mathrm{pF}$	Excitatory external input strength to I neurons
$W^{\mathrm{SX}}_{\mathrm{exc}}$	$1.78\ \ \mathrm{pF}$	Excitatory external input strength to S neurons
$W^{\mathrm{HX}}_{\mathrm{exc}}$	$1.78\ \ \mathrm{pF}$	Excitatory external input strength to H neurons

Table S10. External input parameters.

Parameter mismatches				
**Name**	**Text/Table**	**Source code**	**Description**	**Replicability issue**
A_{inh}	$-/10^{-5}$ AHz	$1\ \mathrm{pF\ ms}$	Amplitude of inhibitory plasticity	Inconsistency
A_{LTD}	$-/0.0014\ \frac{\mathrm{pA}}{\mathrm{mV}^2}$	$0.00014\ \frac{\mathrm{pF}}{\mathrm{mV}}$	LTD amplitude of excitatory plasticity	Inconsistency
V_{peak}	$20\ \mathrm{mV}/-$	$20\ \mathrm{mV}$	Spike threshold of excitatory neurons	Easily missed (only in text)
$r^{\mathrm{E}}_{\mathrm{exc1}}$	$18/4.5\ \mathrm{kHz}$	$22.5\ \mathrm{kHz}$	Rate of sequential external excitatory input to E neurons	Inconsistency
$r^{\mathrm{S}}_{\mathrm{exc}}$	$0{-}10\ \mathrm{kHz}/-$	$10\ \mathrm{kHz}$	Rate of external excitatory supervisor input to S neurons	Inaccuracy
$r^{\mathrm{S}}_{\mathrm{base}}$	$-/-$	$1\ \mathrm{kHz}$	Baseline rate of external excitatory input to S neurons	Missing
$W^{\mathrm{EX}}_{\mathrm{inh}}$	$2.4/1.6\ \mathrm{pF}$	$0.8\ \mathrm{pF}$	Inhibitory external input strength to E neurons	Inconsistency
$W^{\mathrm{IX}}_{\mathrm{exc}}$	$2.4/1.52\ \mathrm{pF}$	$1.52\ \mathrm{pF}$	Excitatory external input strength to I neurons	Inconsistency
$W^{\mathrm{SX}}_{\mathrm{exc}}$	$-/1.6\ \mathrm{pF}$	$1.78\ \mathrm{pF}$	Excitatory external input strength to S neurons	Inconsistency
$W^{\mathrm{HX}}_{\mathrm{exc}}$	$-/1.6\ \mathrm{pF}$	$1.78\ \mathrm{pF}$	Excitatory external input strength to H neurons	Inconsistency
d	$-/-$	$0.1\ \mathrm{ms}$	Synaptic delay	Missing
$d_{\mathrm{u,v}}$	$-/-$	$0\ \mathrm{ms}$	Delay for processing the low-pass filtered membrane potentials	Missing
dt	$0.1\ \mathrm{ms}/-$	$0.1\ \mathrm{ms}$	Resolution time	Easily missed (only in text)
V_0	$-/-$	$[-60, -52]\ \mathrm{mV}$	Initial membrane potential	Missing
u_0, v_0	$-/-$	Julia: $0\ \mathrm{mV}$ MATLAB: $[-60, -52]\ \mathrm{mV}$	Initial low-passed filtered membrane potential	Missing
a^{E}_0	$-/-$	$0\ \mathrm{mV}$	Initial adaption current	Missing
$V^{\mathrm{E}}_{\mathrm{T},0}$	$-/-$	$-52\ \mathrm{mV}$	Initial adaptive threshold	Missing

Table S11. Includes omitted parameters, inconsistencies between original paper and reference implementation, as well as parameters that can easily be missed.

REFERENCES

Clopath, C., Büsing, L., Vasilaki, E., and Gerstner, W. (2010). Connectivity reflects coding: a model of voltage-based stdp with homeostasis. *Nature neuroscience* 13, 344–352. doi:10.1038/nn.2479

Maes, A., Barahona, M., and Clopath, C. (2020). Learning spatiotemporal signals using a recurrent spiking network that discretizes time. *PLOS Computational Biology* 16, 1–26. doi:10.1371/journal.pcbi.1007606

Vogels, T. P., Sprekeler, H., Zenke, F., Clopath, C., and Gerstner, W. (2011). Inhibitory plasticity balances excitation and inhibition in sensory pathways and memory networks. *Science* 334, 1569–1573. doi:10.1126/science.1211095
